# The Skin‐Lightening Efficacy and Mechanisms of Botanical Extracts: A Comprehensive Review

**DOI:** 10.1111/jocd.71213

**Published:** 2026-09-28

**Authors:** Siting Yang, Mingning Ding, Chenglong Ye, Jingning Shen, Lihua Zhang, Anquan Yang, Lihao Gu

**Affiliations:** ^1^ State Key Laboratory for Development and Utilization of Forest Food Resources, Joint Laboratory of Advanced Biomedical Materials (NFU‐UGent), Jiangsu co‐Innovation Center of Efficient Processing and Utilization of Forest Resources Nanjing Forestry University Nanjing China; ^2^ Osman Biological co., Ltd. Huzhou China

## Abstract

**Background:**

Skin pigmentation is regulated by a complex network involving melanogenesis, melanosome transfer, oxidative stress, environmental stimuli, and epidermal turnover. Botanical extracts and phytochemicals have attracted increasing interest as potential skin‐lightening agents. This review evaluates their efficacy, mechanisms, and safety.

**Methods:**

A comprehensive literature review was conducted to synthesize evidence on the skin‐lightening effects and mechanisms of botanical extracts and their bioactive constituents. Evidence was organized into six major pathways: attenuation of ultraviolet and environmental damage; regulation of the microphthalmia‐associated transcription factor (MITF) pathway; tyrosinase (TYR) inhibition; inhibition of melanosome transfer; modulation of redox processes involved in melanin formation; and regulation of epidermal renewal.

**Results:**

The reviewed literature indicates that botanical extracts and phytochemicals can regulate pigmentation through multiple complementary mechanisms. Polyphenols, flavonoids, terpenoids, alkaloids, and other plant‐derived constituents have been reported to influence the MITF/TYR/TRP axis, melanosome transport, oxidative stress, and pigment metabolism. These findings suggest that botanical skin‐lightening activity extends beyond direct TYR inhibition and may involve coordinated regulation of pigment homeostasis. However, evidence strength varies among botanical materials, and much of the available evidence is derived from in vitro, cellular, or animal models. Safety concerns, including melanocytotoxicity and potential leukoderma, also require careful consideration.

**Conclusion:**

Botanical extracts show promise as multifunctional skin‐lightening agents. Future research should emphasize standardized characterization, mechanism‐based evaluation, comprehensive safety assessment, and validation in human skin and clinical settings.

## 1 Introduction

The latest data shows that the global skin‐lightening agent market will grow at a rate of 5.3% from 2024 to 2030 [1]. The related products have a promising future in regions such as Asia, the Middle East, and Africa. China, a major consumer market for brightening products, with its growth in industry size from 60.74 billion yuan in 2023 to an estimated 108.7 billion yuan in 2028 [2] Skin brightening, as a universal dermatological demand, addresses multifactorial pigmentation dysregulation through three primary mechanisms: (1) cutaneous radiance enhancement via stratum corneum light refraction optimization; (2) amelioration of epidermal melanin accumulation through melanogenesis pathway modulation; and (3) preventive mitigation of pigmentary disorders including solar lentigines, melasma, and senile lentigines [3].

## 2 Physiological Basis of Skin Pigmentation

### 2.1 Physiological Functions of Melanin, Melanocytes, and Epidermal Melanin Units

Melanocytes are the primary cells responsible for melanin synthesis and the formation of melanosomes in the skin, and they are mainly distributed in the basal layer of the epidermis and in hair follicles. Within the epidermis, a single melanocyte can establish connections with approximately 40 neighboring keratinocytes via its dendrites, together forming the epidermal melanin unit; this structure serves as a vital foundation for maintaining the distribution of pigment in the epidermis and the coordination of local functions [4]. Skin pigmentation depends primarily on melanin production, as well as the transfer of mature melanosomes to keratinocytes and their subsequent processing [5]. Upon stimulation by ultraviolet radiation, hormones, inflammatory mediators, or local cellular signaling, melanocytes initiate the melanin synthesis process and deliver mature melanosomes to keratinocytes via their dendrites; the keratinocytes carrying these melanosomes gradually migrate upwards as the epidermis differentiates [6]. Once inside keratinocytes, melanosomes can form a supranuclear melanin cap above the cell nucleus, thereby absorbing and dissipating radiant energy and reducing photodamage to the nucleus and genetic material. Consequently, melanin synthesis and melanosome transport not only determine the formation of visible pigmentation but also constitute an important endogenous photoprotective system in the skin.

In addition to their classic roles in pigment formation and photoprotection, melanocytes and the epidermal melanocytic unit in which they reside are involved in the regulation of a broader range of skin homeostasis. Melanocytes do not function in isolation but are an integral part of the epidermal cellular communication network. In addition to establishing structural connections with keratinocytes through dendrites and melanosome exchange, they also engage in bidirectional communication via cell–cell contacts and paracrine signaling, jointly maintaining pigment distribution and local tissue homeostasis [7]. For example, caveolae located at the interface between melanocytes and keratinocytes act as hubs for signal and mechanical regulation, linking signals from neighboring keratinocytes to the remodeling of the melanocyte cytoskeleton, thereby influencing dendrite formation, intercellular contacts, and melanosome transport [8]. Furthermore, melanocytes are capable of sensing inflammation, oxidative stress, and other environmental changes and participate in local intercellular communication and adaptive regulation via immune‐related molecules and soluble mediators [9]. Consequently, melanocytes should be regarded as functional sensory and regulatory cells situated at the skin‐environment interface, and their interactions with keratinocytes, immune cells, and other skin cells collectively form a crucial foundation for maintaining skin homeostasis [10].

The biological role of melanin itself also extends beyond its function as a mere optical barrier. As a complex biopolymer with redox activity, melanin not only absorbs radiation but also participates in electron transfer, free radical scavenging, and the binding of metal ions such as Fe, Cu, and Zn, thereby playing a role in redox buffering and the stabilization of the cellular microenvironment [11]. However, these effects are markedly composition and environment‐dependent. Eumelanin typically exhibits strong light‐absorbing and free‐radical scavenging capabilities, whilst pheomelanin may promote the formation of reactive oxygen species under specific radiation and oxidative conditions. However, excessive or abnormally active melanin synthesis may also be accompanied by the accumulation of reactive intermediates, oxidative stress, and cellular damage. Consequently, intervention strategies targeting melanin production should not be understood simply as inhibiting melanin synthesis but should aim to reduce abnormal pigmentation whilst preserving the melanin system's normal photoprotective, antioxidant, and homeostatic functions.

### 2.2 Classic Melanin Synthesis Signaling Pathways

Melanin synthesis is a process driven by multi‐level signal regulation and a series of enzymatic reactions, in which the microphthalmia‐associated transcription factor (MITF) acts as a central transcriptional hub linking upstream signals to the effector molecules of melanin synthesis, whilst tyrosinase (TYR), as the key rate‐limiting enzyme for tyrosine hydroxylation, plays a crucial role in melanin synthesis. TYR first catalyzes the hydroxylation of L‐tyrosine to form L‐dopa (L‐DOPA) and subsequently oxidizes L‐dopa to dopaquinone (DQ) [12]. In the presence of sufficient cysteine, DQ can form intermediates such as cysteinyl‐dopa with cysteine, and through subsequent oxidation and polymerization, these form sulfur‐containing melanin ranging in color from yellow‐red to reddish‐brown to black, whereas in the absence of cysteine, DQ undergoes intramolecular cyclization to form the important intermediate dopachrome. Dopachrome can undergo spontaneous decarboxylation to Form 5,6‐dihydroxyindole (DHI), which is subsequently oxidized and polymerized to form true melanin, with DHI as the primary structural unit. Under the action of TRP‐2 (also known as dopachrome tautomerase, DCT), dopachrome undergoes tautomerization to Form 5,6‐dihydroxyindole‐2‐carboxylic acid (DHICA), which can be further oxidized and polymerized to form the light brown DHICA‐melanin [13]. TYRP‐1 is also involved in the synthesis of true melanin and in the stability and functional maintenance of the melanin synthesis system; however, its specific catalytic role in human melanocytes is more complex than in animal models and has not yet been fully elucidated.

MITF is situated upstream of this enzymatic system; its importance lies in its ability to integrate intracellular signals from various sources into the transcriptional output of genes related to melanin synthesis. Classic pathways such as cAMP/PKA, MAPK, Wnt/β‐catenin, and PI3K/Akt do not directly drive melanin synthesis independently of one another but rather jointly regulate MITF expression, transcriptional activity, and protein stability at different levels [14]. In particular, elevated cAMP concentrations activate PKA and its downstream transcriptional regulatory mechanisms, thereby promoting MITF transcription. This is the classical pathway through which melanogenesis‐promoting signals enter the MITF core axis. Meanwhile, the Wnt/β‐catenin signaling pathway promotes MITF expression by stabilizing β‐catenin and enhancing associated nuclear transcriptional activity [15]. In contrast, MAPK signaling primarily regulates the transcriptional activity, phosphorylation status, and protein stability of MITF via pathways such as ERK, p38, and JNK. Its ultimate effects are markedly dependent on the specific signaling pathway and the stimulating conditions. The PI3K/Akt pathway can interact with the MAPK network via nodes such as GSK3β and β‐catenin, thereby regulating the expression, stability, and functional state of MITF in different cellular contexts [16].

As a common transcriptional output node, MITF further regulates melanogenesis‐related genes such as TYR, TYRP‐1, and TRP‐2, enabling the various upstream signals to ultimately translate into changes in melanin synthesis capacity. In addition to directly controlling the melanin enzyme system, MITF is also involved in regulating genes associated with the formation, maturation, and intracellular transport of melanosomes. For example, MITF can influence the expression of the melanosome structural protein Pmel17 [17] and is involved in regulating Rab27a, which is associated with melanosome transport [18].

### 2.3 Hormonal Regulation of Melanin Synthesis

Melanin synthesis is not determined solely by tyrosinase activity but is jointly regulated at multiple levels—systemic, tissue, cellular, and subcellular. In addition to light exposure and the local cellular microenvironment, systemic endocrine signals, hormone‐like mediators produced locally in the skin, and autocrine and paracrine regulation can all alter the functional state of melanocytes. The various regulatory factors do not act independently in a simple linear sequence but rather interact through intersecting and partially overlapping signaling networks to jointly influence MITF and its downstream melanogenic enzyme system. The final state of melanin synthesis in melanocytes is, in fact, the result of the combined effects of melanogenic signals, inhibitory signals, substrate supply, and the local tissue environment [19].

Melanocortin derived from POMC is one of the most extensively studied hormonal regulators of melanogenesis; both α‐melanocyte‐stimulating hormone (α‐MSH) and adrenocorticotropic hormone (ACTH) act on the melanocortin 1 receptor (MC1R) on the surface of melanocytes, promoting melanogenesis and favoring the formation of eumelanin. In addition to circulating sources, these peptides can also be produced by local skin cells, thereby forming a regulatory pattern that combines endocrine, paracrine, and autocrine mechanisms [20]. Hormonal regulation does not simply promote melanin production unidirectionally, and certain endocrine or local hormone‐like signals may exert a negative regulatory effect on melanin production by antagonizing melanocortin signaling, altering receptor activity, or modulating MITF stability, thereby maintaining a dynamic equilibrium between pro‐pigment and anti‐pigment signals [21].

Apart from the melanocortin system, sex hormones represent another important class of regulatory factors linking systemic endocrine status to changes in skin pigmentation. Pigment changes associated with pregnancy, oral contraceptives, and the female reproductive cycle, as well as the high prevalence of melasma in women, all suggest that estrogen and progesterone signaling are involved in the regulation of pigment homeostasi [22]. Among these, the stimulatory effect of estrogen on melanocyte function and pigment production has been supported by a considerable body of research, whilst the role of progesterone is more cell‐type and environment‐dependent. Sex hormones do not determine pigmentation in isolation but rather act in synergy with factors such as light exposure, oxidative stress, inflammation, and changes in the vascular and dermal microenvironment. Therefore, conditions such as melasma are best understood as an imbalance in pigment homeostasis driven by a combination of local and environmental factors against a background of hormonal susceptibility, rather than as a result of elevated levels of a single sex hormone [23]. Furthermore, systemic endocrine and metabolic signals—such as glucocorticoids, thyroid‐related hormones, growth hormone, and IGF‐1—are also thought to regulate melanocyte proliferation, differentiation, metabolism, and stress responses. These signals intersect with the MITF regulatory network via pathways such as MAPK and PI3K/Akt, thereby influencing melanin production indirectly or in a context‐dependent manner [24].

In addition to the classic regulation by melanin and sex hormones, melatonin has also attracted attention in recent years as a regulator of skin pigmentation. Melatonin should not be understood solely as a circadian hormone produced by the pineal gland; the skin itself possesses a complete melatonin system. Keratinocytes, melanocytes, fibroblasts, hair follicle cells, and other cells can all participate in the tryptophan–5‐hydroxytryptamine–melatonin metabolic pathway, producing bioactive melatonin and its metabolites [25]. Consequently, the skin's melatonin system is better understood as a local regulatory system involved in antioxidant defense, anti‐inflammation, photoprotection, and the maintenance of homeostasis, rather than merely as a skin‐lightening factor that affects skin tone. From the perspective of pigment regulation, the relationship between melatonin and melanin production is clearly context‐dependent. Studies have found that UVB can induce melanocyte stress, p53 activation, and increased TYR expression, whilst melatonin treatment can reduce UVB‐induced p53 activation and pigmentation, suggesting that its role in pigment regulation relies more on improving the photodamage response and cellular homeostasis than on directly inhibiting tyrosinase activity [26].

The melanin production system is not only controlled by exogenous hormones; its own metabolic state can also participate in regulating the final pigment output, exhibiting certain characteristics of endogenous feedback regulation. Although L‐tyrosine and L‐DOPA are sequential substrates and intermediate products in the melanin biosynthetic pathway, their participation in the reaction is not entirely passive. Previous studies have suggested that both also possess hormone‐like or bioregulatory effects, capable of influencing melanocyte function, the expression of melanin‐synthesis‐related proteins, as well as melanosome formation and tyrosinase processing [27]. In addition to classic systemic hormones, the skin itself possesses a local neuroendocrine regulatory system; the neuropeptides, neurotransmitters, and hormone‐like mediators produced by this system can jointly regulate melanocyte function alongside immune and paracrine signals.

### 2.4 The CRH‐POMC Neuroendocrine Axis in the Regulation of Skin Pigmentation

Skin pigmentation depends not only on the MITF‐tyrosinase complex within melanocytes but is also regulated by upstream local stress and neuroendocrine signaling. The skin itself is capable of expressing corticotropin‐releasing hormone (CRH), CRH receptors, and POMC‐related components, thereby forming a neuroendocrine system with local autonomous regulatory capacity [28].

The CRH‐POMC neuroendocrine axis is a key system linking local stress responses in the skin to pigment regulation. CRH, situated relatively upstream, mediates responses in different skin cell types via CRHR1 and CRHR2 and further influences the expression and function of the POMC system [29]. CRH signaling in the skin is not a simple replication of the central hypothalamic–pituitary–adrenal axis but exhibits marked dependence on cell type, receptor subtype, and the microenvironment. POMC is the central precursor molecule of this axis. POMC itself is not a single effector hormone, but requires tissue and cell‐type‐specific cleavage by prohormone‐converting enzymes, particularly processing systems such as PC1/3 and PC2, to form various bioactive peptides including ACTH, α‐MSH, β‐MSH, γ‐MSH, β‐lipokine and β‐endorphin [30]. Different cells possess varying capacities for processing POMC; consequently, an increase in POMC expression levels does not necessarily imply a synchronous increase in the various downstream peptides at a fixed ratio. The ultimate biological effects depend on the local composition of processing enzymes, cell type, and tissue state; this plasticity enables the POMC system to generate signaling molecules with distinct receptor selectivity and functional characteristics from a single precursor.

Among the various POMC‐derived peptides, α‐MSH and ACTH are currently the two classes of effector peptides with the most clearly established relationship to skin pigment regulation. Both act on the MC1R on the surface of melanocytes, constituting the main pathway through which the POMC system transmits pigment‐promoting signals to melanocytes [31]. In contrast, β‐endorphin can influence melanocyte proliferation, differentiation, dendrite formation, and melanin production via the opioid receptor system, including the μ‐opioid receptor [32]. Keratinocytes and melanocytes, among others, are also capable of locally producing POMC‐derived peptides, endowing this system with distinct autocrine and paracrine characteristics [33]. Consequently, the regulation of the pigment system by POMC cannot be simply equated with α‐MSH‐MC1R signaling, but should be understood as a multi‐pathway regulatory network formed jointly by different POMC processing products via their respective receptors.

### 2.5 Neuro‐Immune‐Endocrine Regulatory Networks in Skin Pigment Homeostasis

Skin pigmentation is not the result of the tyrosinase system within melanocytes operating independently; rather, it is an adaptive homeostatic process jointly regulated by neural, endocrine, and immune signals in response to changes in the external environment perceived by the skin. As the body's largest organ for sensing the environment, the skin is constantly exposed to a variety of external factors, including ultraviolet radiation, oxidative stress, pollutants, microbial stimuli, mechanical damage, and inflammatory environments. These stimuli are recognized collectively by keratinocytes, sensory nerve endings, and immune‐related cells and are converted into local regulatory signals via intercellular communication, thereby further activating the skin's neuro‐immune‐endocrine regulatory network. Taking post‐inflammatory hyperpigmentation (PIH) as an example, its development involves not only inflammation‐induced overactivation of melanocytes and enhanced melanin production but also the disruption of the homeostasis of the keratinocyte‐melanocyte unit by the inflammatory environment, as well as abnormal distribution of melanosomes [34]. Therefore, hyperpigmentation should not be understood as an abnormality in a single melanin production pathway, but rather as a comprehensive biological response in which the skin responds to environmental stress and maintains tissue homeostasis.

The sensory nervous system serves as a key gateway through which environmental information enters the skin's regulatory network. Upon exposure to external stimuli, sensory nerve endings in the skin release various neurotransmitters and neuropeptides, including substance P, calcitonin gene‐related peptide (CGRP), vasoactive intestinal peptide (VIP), and neuropeptide Y (NPY). These signals not only participate in the regulation of neuroinflammation but also influence the functional state of keratinocytes, immune cells, and melanocytes, making the nervous system a crucial information transmission pathway linking environmental stimuli to cellular responses. At the same time, signals of neural origin can interact with the local neuroendocrine system of the skin and, through neuropeptides, CRH/POMC‐related signaling, and other hormone‐like mediators, influence melanocyte proliferation, differentiation, stress responses, and the maturation of melanosomes [35].

The immune system, in turn, serves as a crucial regulatory link between environmental stimuli and melanocyte behavior. Keratinocytes, immune cells, and melanocytes themselves can all produce cytokines, chemokines, and immunomodulatory molecules, collectively participating in local inflammatory and tissue repair processes. Melanocytes are not only capable of expressing pattern recognition receptors and immune‐related molecules but can also detect pathogen‐associated molecular patterns (PAMPs), damage‐associated molecular patterns (DAMPs), and oxidative stress signals and participate in immune regulation by releasing IL‐1, IL‐6, TNF‐α, IFN‐related factors, and chemokines [36]. At the same time, immune signals such as IFN‐γ and TNF‐α can in turn regulate melanocyte adhesion, survival, differentiation, and chemokine expression, maintaining a dynamic equilibrium between immune defense and tissue damage. Recent studies have found that CLEC12B can regulate IFN‐γ‐induced expression of CXCL9, CXCL10, and CXCL11 via the STAT1/IRF1 signaling pathway, further demonstrating that melanocytes can actively participate in local immune signaling [37].

Furthermore, as an indole signaling molecule with neuroendocrine properties, melatonin is also involved in the regulation of the skin's neuro‐immune‐endocrine network. Melatonin exerts its effects via MT1/MT2 receptors, AhR, PPARγ, and non‐receptor‐dependent pathways, regulating ROS scavenging, Nrf2‐mediated antioxidant defense, the NF‐κB inflammatory pathway, mitochondrial homeostasis, MMP expression, and apoptosis [38].

Under the combined influence of neural, endocrine, and immune signals, melanocytes exhibit a dynamically regulatable functional state, encompassing processes such as melanin synthesis, melanosome formation and transport, cell proliferation and differentiation, and stress adaptation, thereby determining the final level of pigment output. Under normal conditions, this multi‐level network enables the skin to maintain dynamic coordination between environmental adaptation, immune defense, and pigment balance; however, when environmental stimuli persist or there is an imbalance in neural, immune, and endocrine regulation, this may lead to abnormal pigmentation or hypopigmentation. Consequently, skin pigment homeostasis is essentially the result of the continuous integration of environmental signals and local regulatory networks; its maintenance does not rely on unidirectional signal transmission but rather achieves a dynamic equilibrium through bidirectional communication and feedback regulation between the nervous, endocrine, and immune systems and melanocytes.

### 2.6 Ultraviolet Radiation and Skin Pigment Homeostasis

Ultraviolet radiation (UVR) is one of the key environmental factors influencing skin pigment homeostasis. Its effect is not simply manifested as an increase in melanin due to damage, but rather as a complex process resulting from the skin's perception of light stress, involving the photodamage response, neuroendocrine regulation, immune responses, and adaptive protective mechanisms of the melanocytic system [39]. Excessive or prolonged UV exposure can lead to hyperpigmentation, photoageing, and an increased risk of skin cancer; however, moderate UV‐induced melanin production is itself an important adaptive response that helps the skin maintain photoprotection.

There are marked differences in the mechanisms of action of different UVR bands within the skin. UVA (320–400 nm), which accounts for over 90% of surface UV radiation, has the ability to penetrate deep into the dermis, where it induces the production of reactive oxygen species (ROS) via photochemical reactions, leading to oxidative stress, mitochondrial damage, matrix degradation, and alterations in the dermal microenvironment; it is a major driver of photoageing [40]. In contrast, UVB (280–320 nm) primarily acts on the epidermis, where it is directly absorbed by DNA and induces DNA photodamage such as cyclobutane pyrimidine dimers (CPDs) and 6,4‐photoproducts, subsequently activating the p53‐mediated damage response pathway [41].

UVB‐induced delayed pigmentation is currently one of the best‐characterized photoadaptive responses. UVB first acts on keratinocytes, causing DNA damage and activating p53 signaling, which subsequently promotes the production of POMC‐related peptides. Among these, α‐MSH and ACTH act on melanocytes via paracrine mechanisms, activating MC1R‐related melanogenic signals to promote melanin production and photoprotective adaptive response [31]. This process reflects the functional coupling between keratinocytes and melanocytes. That is, melanin production is not carried out independently by melanocytes but rather constitutes a protective response initiated by the skin upon sensing photodamage. The melanin produced is subsequently transported into keratinocytes via melanosomes, where it forms a supranuclear melanin cap above the cell nucleus, thereby absorbing and dissipating ultraviolet radiation and reducing the risk of further DNA damage. However, UV‐induced pigmentation is not entirely synonymous with the synthesis of new melanin. Whilst UVB primarily induces delayed melanogenesis, UVA tends to cause immediate pigmentation deepening through the oxidation of existing melanin, the generation of reactive oxygen species (ROS), and the redistribution of melanin granule [42]. The persistent pigmentation changes induced by UVA may involve oxidative stress, the release of inflammatory mediators, and alterations in the dermal microenvironment [31].

From a physiological perspective, UV‐induced melanin production exhibits distinct adaptive characteristics. The melanin system reduces further UV damage by enhancing light absorption capacity, representing a crucial defense mechanism developed through the long‐term evolution of the skin. However, when UV exposure exceeds the skin's regulatory capacity, persistent DNA damage, ROS accumulation, and the activation of immune and inflammatory signals can lead to photoageing, post‐inflammatory hyperpigmentation, and abnormal pigmentation. The multifactorial etiology of PIH encompasses viral infections, contact dermatitis, procedural trauma (e.g., surgical/chemical injuries), and intrinsic factors like Fitzpatrick skin types IV‐VI exhibiting post‐acne PIH predisposition [43]. The Fitzpatrick skin classification is presented in Table 1 [43]. Mechanistic studies reveal that PIH pathogenesis involves dual pathways: (1) inflammation‐triggered melanocyte hyperactivity with excessive melanosome production and (2) impaired melanin dispersion through disrupted keratinocyte‐melanocyte unit homeostasis [44].

**TABLE 1:** The fitzpatrick skin types [43].

Skin Type	Sunburn and Tanning
I	Burns easily, never tans
II	Burns easily, tan minimally with difficulty
III	Burns moderately, tans moderately and uniformly
IV	Burns minimally, tans moderately and easily
V	Rarely burns, tans profusely
VI	Never burns, tans profusely

Among clinical pigmentary disorders, melasma exemplifies the complexity of long‐term interactions between UV radiation and the skin microenvironment. Melasma is not caused solely by an increase in epidermal melanin but rather involves the combined action of multiple factors, including melanocyte activation, damage to the basement membrane, dermal photoageing, vascular abnormalities, inflammatory responses, and changes in hormone levels [23]. Chronic UV exposure alters the dermal environment through reactive oxygen species (ROS) generation, increased expression of matrix metalloproteinases (MMPs), and extracellular matrix degradation, thereby promoting the persistence of pigmentation. Furthermore, visible light‐particularly high‐energy visible light (HEVL, 400–450 nm) and UVA1 (370–400 nm)‐is also thought to contribute to the exacerbation of melasma. HEVL can activate relevant signaling pathways via the melanocyte photoreceptor molecule Opsin‐3, promoting tyrosinase expression and pigment formation, a phenomenon that is particularly pronounced in individuals with darker skin tone [45]. It is worth noting that not only does melatonin play a role in the regulation of UV‐induced oxidative stress, but its metabolites, AFMK and AMK, also possess free radical‐scavenging and cell‐protective properties, and thus constitute an important component of the skin's defense system against photodamage [46]. Consequently, modern photobiological research is no longer limited to UVB but must comprehensively consider the effects of UVA, UVA1, visible light, and the oxidative‐inflammatory microenvironment on pigment homeostasis.

Based on the above mechanisms, photoprotection should not only focus on reducing UV‐induced damage but also on preserving the skin's normal phototolerance. Traditional SPF primarily reflects UVB protection capacity, whilst failing to adequately evaluate the protective effects against pigmentation associated with UVA and visible light. Consequently, for individuals with melasma and light‐sensitive pigmentary disorders, protective strategies offering broad‐spectrum UVA/UVB protection and covering visible light are more highly recommended; for example, colored sunscreens containing iron oxide can reduce the risk of light‐induced pigmentation deepening by absorbing HEVL. At the same time, research into plant‐derived active ingredients for preventing hyperpigmentation should not focus solely on tyrosinase inhibition. The aim of skin‐lightening interventions is not to completely block melanin production but rather to reduce abnormal pigmentation whilst maintaining the normal photoprotective function of the melanin system.

## 3 Research on Natural Compound‐Derived Skin‐Lightening Agents

### 3.1 Reduction of Sun Exposure Signals and External Damage

Botanical extracts mitigate UV‐induced and environmentally stressed melanin hyperpigmentation through multi‐target mechanisms, with their protective mechanisms categorized into two classes: direct ultraviolet radiation blocking and post‐damage repair. Scutellariae Radix and Flos Sophorae Immaturus have been documented to exhibit superior absorption capacities against UVB (280–315 nm) and UVA (315–400 nm) respectively [47]. The ethanol‐soluble fraction (butanol extract) of Scutellariae Radix demonstrates significant UV radiation attenuation, achieving a Sun Protection Factor (SPF) of 22.7 at 5% formulation concentration in vitro testing. Chrysanthemum extract exhibits broad‐spectrum UV absorption across 200–400 nm wavelengths, with its UV absorbance coefficient demonstrating a linear correlation to concentration, which was attributed to its chlorogenic acid content [48]. Notably, Chrysanthemum achieves > 90% UV absorption in both UVB (280–315 nm) and UVC (100–280 nm) bands [47], and in combination with Scutellariae Radix enables full‐spectrum coverage across the UVB range [48]. Saussurea involucrata cultures have been reported to effectively block 280–400 nm UV radiation, potentially mediated by its flavonoid constituents and flavonoid‐mediated photoprotective pathways [49].

Natural polysaccharides are widely recognized for their multifunctional dermatological properties, including humectant, antioxidant, and skin barrier restorative capacities [50]. In cell cultures and experimental models, the enzymatic hydrolysis of polysaccharide components from purslane can alleviate UVB‐induced photodamage and exerts a protective effect by reducing oxidative stress, apoptotic cascades, and inflammatory responses [51]. Similarly, polysaccharide pretreatment derived from Cordyceps exhibits significant attenuation of UVB‐triggered photoproducts, cyclobutane pyrimidine dimers (CPDs), formation, suggesting its potential as a photoprotective adjuvant [52].

In addition to the ultraviolet rays, other environmental aggressors, such as air pollution and lifestyle, can induce oxidative stress in human skin cells. Oxidative stress promotes the release of α‐MSH from keratinocytes, which promotes melanogenesis through paracrine action. Coenzyme Q10, a naturally occurring antioxidant in humans, has multiple protective effects on keratinocytes and melanocytes, which target and activate the nrf2/ARE antioxidant pathway to inhibit the production of α‐MSH and effectively improve the following melanogenesis [53]. Similarly, in a mouse model of melasma, Panax notoginseng root polysaccharides demonstrated efficacy in improving pigmentation through dual regulation of the Nrf2/Keap1 redox axis‐upregulating nuclear factor erythrocyte‐2‐related factor 2 (Nrf2) whilst simultaneously inhibiting the activity of Kelch‐like ECH‐associated protein 1 (Keap1). However, these findings are primarily based on evidence from animal models, and their clinical relevance in human melasma requires further validation [54].

In PIH management, targeted intervention effectively counteracts trauma‐induced melanogenesis amplification. Soybean‐derived isoflavones exhibit a distinct mechanism: Genistein (4′,5,7‐trihydroxyisoflavone) attenuates UVB‐induced erythema via COX‐2/prostaglandin E2 (PGE2) axis inhibition [55] and 6,7,4′‐Trihydroxyisoflavone mitigates photodamage through matrix metalloproteinase‐1 (MMP‐1) suppression [56]. These compounds represent novel skin‐lightening agents. In the B16 melanoma cell model and the guinea pig skin model, the liquorice flavonoid glycyrrhetinic acid exhibited dual anti‐melanogenic and anti‐inflammatory effects, and structural analysis revealed that its biphenyl hydroxyl group mediates the inhibition of melanin synthesis, although its clinical relevance in humans requires further verification [57]. In a study on solanine in Australia, the compound was found to inhibit UVB‐activated p38 MAPK phosphorylation and NF‐κB nuclear translocation, thereby reducing the release of pro‐inflammatory factors such as IL‐1α, IL‐1, IL‐8 and IFN‐γ, and consequently attenuating the inflammatory stimulation of melanocytes by keratinocytes [58].

### 3.2 Regulating the Melanin Synthesis Signaling Pathway in the MITF Center

Botanical extracts exhibit multi‐target modulatory effects on melanogenesis pathways, primarily through transcriptional modulation of melanogenic genes such as microphthalmia‐associated transcription factor (MITF), the “master regulator” of melanin synthesis, thereby suppressing transcriptional activation of the transcriptional orchestration of melanogenic enzymatic systems (tyrosinase, TRP‐1, TRP‐2). The hierarchical regulatory network of MITF, governing pigment cell differentiation and melanosome biogenesis, presents an ideal platform for plant‐derived multi‐component synergism.

Artemisia argyi leaves ethanol extract inhibits paracrine products (SCF, ET‐1) from keratinocytes from activating the MITF/TYR pathway, effectively suppressing melanogenesis in melanocytes [59]. The main components of Artemisia argyi include volatile oils, flavonoids, and polysaccharides [60]. The flavonoid scaffold, characterized by two phenolic hydroxyl‐bearing benzene rings connected via a three‐carbon alkyl chain (C6‐C3‐C6 configuration), is recognized for its capacity to form hydrogen bonds with target proteins through the phenolic‐OH groups. This structure–activity relationship (SAR) explains flavonoids' established roles as potent skin‐lightening agents with multi‐target therapeutic potential [61].

Tea polyphenols, recognized as potent antioxidant agents, have been demonstrated through purified extracts to exert suppressive effects on the intracellular melanogenic pathway within melanocytes. The isolation of specific catechin derivatives from tea polyphenol extracts, namely epigallocatechin gallate (EGCG), epicatechin gallate (ECG), and gallocatechin gallate (GCG), has revealed their targeted inhibition of melanogenesis‐related biomarkers, with distinct mechanistic profiles. EGCG exhibits the most pronounced suppression of MITF (microphthalmia‐associated transcription factor) gene expression, while ECG shows superior inhibitory activity against melanogenic pathway master regulators TYR (tyrosinase) gene transcription [62]. Notably, these compounds collectively demonstrate significant downregulation of key melanogenic proteins in melanocytes, including phosphorylated cAMP response element‐binding protein 1 (p‐CREB1), MITF, TYR, tyrosinase‐related protein 1 (TRP‐1), and TRP‐2 [62]. Structural analysis suggests that the galloyl moiety common to these catechin derivatives serves as the primary pharmacophore responsible for their tyrosinase inhibitory activity, as evidenced by the consistent anti‐melanogenic efficacy across gallic acid‐containing catechins. Comparatively, floral extracts from Camellia oleifera Abel demonstrate superior inhibitory potency against MITF and its downstream enzymatic targets (TYR, TRP‐1, TRP‐2) relative to equivalent concentrations of kojic acid (KA) [63]. Parallel findings from Scutellaria baicalensis extracts reveal that its flavonoid components, baicalein and baicalin, significantly suppress MITF protein expression through epigenetic modulation [64]. These findings are primarily based on cell‐based experiments, but their clinical relevance in humans requires further verification. These cumulative findings underscore the multifaceted regulatory capacity of plant‐derived flavonoids across the melanogenic axis, spanning transcriptional control (MITF), enzymatic inhibition (TYR), and post‐translational modification (TRP‐1/2), positioning them as promising candidates for hyperpigmentation therapeutics.

Flavonoid derivatives also exhibit significant regulatory effects on melanogenesis‐associated gene expression. UVB irradiation (280–315 nm) activates c‐Jun N‐terminal kinase (JNK) phosphorylation and p38 signaling in normal human melanocytes (NHMs), subsequently enhancing mitogen‐ and stress‐activated kinase 1 (MSK1)/cAMP response element‐binding protein (CREB) phosphorylation cascades, thereby upregulating downstream MITF and EDNRB melanogenic transcriptional networks. This UVB‐induced melanogenic activation is effectively suppressed by the MSK1 inhibitor H89 and Pinus maritima bark extract (PBE) [65]. Notably, combinatorial UVB exposure and daily progesterone injections in a murine melasma model demonstrate zinc glycyrrhizinate‐mediated suppression of hyperpigmentation through targeted inhibition of JNK/p38 mitogen‐activated protein kinase (MAPK) phosphorylation [66]. Through network pharmacology screening and molecular docking validation, Petanin‐an acylated anthocyanin isolated from *Solanum tuberosum* L.‐has been identified as a potent inhibitor of ERK/ribosomal S6 kinase (RSK) phosphorylation in zebrafish ( *Danio rerio* ) models, concurrently suppressing CREB/MITF‐associated gene transcription and translation [67]. Paradoxically, this inhibitory activity coincides with JNK phosphorylation activation, contrary to the established conception of JNK‐mediated melanogenesis stimulation, warranting further mechanistic exploration. Other flavonoid derivatives, including norartocarpetin from *Artocarpus* species, demonstrate cross‐species anti‐melanogenic efficacy through JNK/p38/CREB/MITF pathway modulation, as validated in B16F10 melanoma cells, human dermal fibroblasts (Hs68 cell line), and BALB/c nude mice [68]. Hot‐water extracts of *Torreya nucifera* enriched with epigallocatechin gallate (EGCG) exhibit dose‐dependent suppression of B16F10 melanogenesis through JNK/p38 targeting, coupled with favorable safety profiles [69]. Sodium houttuyfonate (chemically defined as dodecanoylacetaldehyde sodium bisulfite adduct), a synthetic derivative of houttuynin via bisulfite addition, has been identified through high‐throughput screening as a potent melanogenesis inhibitor in B16F10 and Melan‐a murine melanocyte models. Its anti‐melanogenic mechanism involves dual‐pathway modulation: suppression of p38/JNK MAPK phosphorylation cascades and inhibition of NF‐κB nuclear translocation, showing the cross‐talk between inflammatory and pigmentary signaling [70]. This effect may be mainly due to the hydrogen bonding of its sulfite groups.

Current evidence indicates that carboxyl‐containing phytochemicals, particularly phenolic acids, exert melanogenesis regulation through melanogenesis pathways. Ginseng ( *Panax ginseng* ) root extracts demonstrate melanin suppression primarily mediated by vanillin derivatives, which target the nitric oxide/protein kinase G (NO/PKG) signaling axis to inhibit CREB phosphorylation [71]. A comparative study of phenolic compounds derived from oregano revealed that the structural differences between vanillic acid (4‐hydroxy‐3‐methoxybenzoic acid) and its aldehyde counterpart, vanillin (4‐hydroxy‐3‐methoxybenzaldehyde), may influence their anti‐melanogenic activity [72], providing mechanistic validation for the role of vanillic acid in ginseng‐mediated melanin synthesis inhibition and establishing the carboxyl group as a key pharmacophore in phenolic skin‐whitening agents.

### 3.3 Directly Inhibits Tyrosinase Activity

Developing direct tyrosinase inhibitors remains the core strategy for plant‐derived skin‐lightening agents. Various plant‐derived tyrosinase inhibitors have been discovered. Evidence has been documented for the tyrosinase inhibitory effects of volatile oil components. Angelica dahurica volatile oil shows reversible non‐competitive inhibition against mushroom tyrosinase and B16 cell tyrosinase [73] Citrus essential oils from *Citrus medica* L., *Citrus medic* L., and *Gannan navel* orange demonstrate non‐competitive inhibition (IC50: 1.62–1.74 mg/mL) against tyrosinase, containing monoterpenes (d‐limonene, γ‐terpinene), sesquiterpenes, alcohols, and aldehydes [74]. These findings suggest that certain volatile plant compounds may exert an inhibitory effect by altering enzyme conformation or influencing the enzyme‐substrate binding environment; however, the current evidence is derived primarily from in vitro enzymatic studies and cellular models, and their actual efficacy in human skin requires further validation.

Tyrosinase inhibition mechanisms include chelating copper ions in the active center, acting as competitive substrates, and altering enzyme 3D structure.

The polyphenolic compound ellagic acid (EA) exerts inhibitory effects on melanogenesis by chelating the copper‐iron active sites of tyrosinase. Pretreatment with the autophagy inhibitor 3‐MA attenuates EA's melanogenesis‐inhibitory effects, indicating that this inhibition is associated with autophagy activation in melanocytes [75]. Compared with vanillin, vanillic acid exhibits superior antioxidant capacity and tyrosinase‐inhibiting activity. This difference in efficacy may be attributed to the carboxyl group of vanillic acid, which enhances metal ion chelation at the copper‐binding site of tyrosinase and forms stable π‐π stacking interactions with the enzyme's hydrophobic pocket [72].

Research has revealed that pterostilbene, a dimethyl derivative of resveratrol derived from blueberries, can be oxidatively metabolized by tyrosinase to form o‐quinone. Notably, the metabolic conversion rate of pterostilbene is several times higher than that of resveratrol (RES). Furthermore, the presence of two lipophilic methyl groups in pterostilbene's structure enhances its membrane permeability and intracellular protein‐binding capacity, which may induce higher cytotoxicity in melanocytes [76]. Another resveratrol analog, oxyresveratrol (a stilbenoid), can be sequentially metabolized by tyrosinase to generate o‐diphenol, o‐quinone, and p‐quinone. However, these reactions require the presence of oxy‐tyrosinase [50]. Researchers screening six natural tyrosinase inhibitors (including kojic acid) found that norartocarpetin from Artocarpus species exhibits the lowest binding energy and forms tighter interactions with tyrosinase [77].

In a screening study of 78 plant species, the rhizome of Gastrodia elata demonstrated significant potential. Consequently, its naturally occurring T1 compound (bis(4‐hydroxybenzyl)sulfide), T2 compound, and derivatives T3‐T5 were selected for tyrosinase inhibition validation and structure–activity analysis. The results showed that T1 inhibits tyrosinase even at low effective concentrations, outperforming arbutin and kojic acid; furthermore, no toxicity was observed in acute oral administration trials in mice, suggesting a certain degree of preliminary safety. However, these findings constitute evidence of acute toxicity in animals only and cannot be directly equated with the safety of long‐term topical use in humans. Comparative analysis of T2–T5 compounds indicated that T1's remarkable tyrosinase inhibition primarily stems from two factors: the strong sulfur‐copper ion interaction potential and high conformational freedom of its benzene‐sulfur linkage [78].

Triterpenoid compounds derived from Poria cocos are also used in research into the structural mechanisms of tyrosinase. The structural basis suggests that these compounds likely alter the three‐dimensional conformation of tyrosinase by modifying its hydrophobic environment, thereby affecting substrate binding [79].

### 3.4 Inhibition of Melanocyte Activity

The proliferation and differentiation dynamics of mammalian epidermal melanocytes are modulated by a spectrum of keratinocyte‐derived paracrine mediators, including α‐melanocyte‐stimulating hormone (α‐MSH), adrenocorticotrophic hormone (ACTH), and growth factors spanning the neurotrophic (nerve growth factor), hematopoietic (granulocyte‐macrophage colony‐stimulating factor (GM‐CSF), leukemia inhibitory factor), and mitogenic (basic fibroblast growth factor, hepatocyte growth factor) families [80]. α‐MSH is one of the commonly used in vitro melanogenesis‐inducing factors and may also be involved in the activation and functional regulation of melanocytes [81].

Multiple natural extracts demonstrating melanocyte inhibitory activity have been identified. In the B16 cell model, Paris polyphylla extract inhibited melanocyte proliferation at a concentration of 800 μg/mL, accompanied by a decrease in melanin‐related parameters [82], while tea polyphenols (e.g., EGCG, ECG, GCG) [62] and *Camellia* flower extract [83], both rich in polyphenolic compounds, effectively inhibit B16 melanoma cell proliferation, with the latter additionally reducing dendritic structure formation in B16 cells. Notably, black tea extract has also been reported to inhibit melanocyte proliferation in cell models [84]. These studies, which are primarily based on the B16 melanoma cell model, suggest a potential for regulating melanocyte activity and morphology; however, there are still differences compared with normal human melanocytes and actual responses in human skin, and further validation is required.

Mechanistic studies on ginsenosides reveal concentration‐dependent effects, whereas ginsenoside Rb1 exhibits anti‐proliferative activity against B16 cells at concentrations > 125 μg/mL [85]. GM‐CSF is a keratinocyte‐derived growth factor that can be induced by UVA and may be involved in the regulation of melanocyte activation and proliferation following UV exposure [86], in mediating ginsenoside‐mediated melanocyte suppression. Both Korean Red Ginseng extract (KRGE) and its saponin fraction (SKRG) significantly downregulate UVB‐induced GM‐CSF expression in SP‐1 keratinocytes [87], while ginsenoside Rh3 inhibits UVB‐triggered GM‐CSF production in SP‐1 cells through suppression of PKCδ and ERK phosphorylation [88].

### 3.5 Inhibition of Melanin Transfer

The primary mechanisms of melanin transfer involve coordinated exocytosis of melanosomes by melanocytes and subsequent endocytosis by keratinocytes. The cytoskeletal protein integrin‐linked kinase (ILK) retains immature melanosomes in the perinuclear regions of melanocytes and ensures stable transport of mature melanosomes along microtubule tracks. Glycogen synthase kinase‐3 (GSK‐3) disrupts microtubule assembly and impairs melanocyte dendriticity, thereby inhibiting melanin transport [89]. Studies on the caffeic acid derivative N‐(4‐methoxyphenyl)caffeamide (K36E) demonstrate its dual action: suppression of GSK3β expression and inhibition of melanogenesis‐related signaling molecules (p‐CREB, MITF, and TRP‐1), positioning it as a promising skin‐lightening agent [90]. However, this study did not explore the impact of K36E on melanosome trafficking; therefore, further mechanistic investigations are warranted to elucidate its role in melanin transport pathways.

Rab11b primarily mediates melanosome membrane remodeling to facilitate keratinocyte‐induced melanocyte exocytosis and subsequent melanosome transfer to keratinocytes [91]. The compound formula Xinqibai (XQB) (containing seven botanical ingredients) systemically suppresses the expression of melanosome transport complex proteins, including Rab27a, Myo5a, and MLPH [92].

The Rab27A‐Myosin Va‐MLPH tripartite complex plays a central role in melanosome transport and distribution [93, 94]. Wogonin, the active component derived from Scutellaria baicalensis, demonstrates dual skin‐lightening effects by not only suppressing melanogenesis through inhibition of melanin synthesis pathways but also disrupting melanosome transfer via calpain/proteasome‐mediated degradation of MLPH protein, a mechanism that operates independently of the suppression of mRNA expression levels for Rab27A, Myosin Va, or MLPH, thereby achieving comprehensive blockade of melanosome translocation to keratinocytes [64]. To elucidate the structural basis of melanosome transport defects, wogonin and its analogs were tested in B16F10 cells. Results identified 8‐O‐methylflavone analogs (including wogonin) as the most potent melanosome transport inhibitors [64]. In contrast, manassantin B from Saururus chinensis Baill. primarily inhibits melanosome transport by disrupting Myosin Va‐MLPH interactions [95]. These studies provide relatively direct cytological evidence that plant‐derived compounds target the melanosome transport complex.

Protease‐activated receptor 2 (PAR‐2) is suggested to regulate melanotransfer due to the regulation of dendritic formation. Inhibition of PAR‐2 resulted in skin lightening of the pigmentation [96]. Treatment with peony seed oil reduced the gene expression levels of PAR‐2, accompanied by a decrease in melanin content [97]. It suggests that it may influence the processes of melanin synthesis and transport; however, its direct effect on melanosome uptake requires further verification. Interestingly, paeonol, as the primary phenolic acid component of peony ( *Paeonia suffruticosa* ), demonstrates melanin transfer inhibition in melanocyte‐keratinocyte co‐culture systems. It significantly reduces SLIGRL‐induced melanin transfer (mediated by PAR‐2 activation) without altering PAR‐2 gene expression levels. This phenomenon may be attributed to paeonol's multifaceted mechanism involving targeted modulation of the JNK/SAPK signaling pathway, suppression of cAMP response element‐binding protein (CREB) phosphorylation at Ser133, and downregulation of MITF‐mediated tyrosinase (TYR) expression, collectively resulting in attenuated melanogenesis and subsequent reduction in melanosome transfer, thereby necessitating systematic investigation to delineate the causal relationships between these molecular events [98].

### 3.6 Enhancement of Melanin Antioxidant Capacity

The antioxidant capacity of plant extracts exhibits a significant positive correlation with their skin‐lightening efficacy, as evidenced by documented skin‐lightening effects of common dermal antioxidants including resveratrol, vitamin C, coenzyme Q10, and green tea extracts. These agents exert multi‐targeted ameliorative effects through three interconnected mechanisms: ameliorating oxidative stress in epidermal cells, reducing intracellular free radical accumulation in melanocytes triggered by oxidative stress, and inhibiting DOPA oxidation during melanogenesis. Furthermore, phytomelatonin is present in plants and is recognized as a class of endogenous plant signaling molecules with broad regulatory functions, involved in processes such as seed germination, root and stem growth, photosynthesis, stomatal regulation, fruit ripening, leaf senescence and stress responses. Under drought, salt stress, low temperature, heavy metal, pesticide, UV radiation, and pathogenic microorganism stress, phytomelatonin can help plants maintain redox homeostasis and stress tolerance by scavenging ROS/RNS, regulating the ascorbic acid/glutathione cycle, activating the antioxidant enzyme system, and interacting with plant hormones such as ABA, ethylene, jasmonic acid, salicylic acid, and brassinosteroids, to help plants maintain redox homeostasis and tolerance to stress [99]. However, the direct effects of plant‐derived melatonin on skin pigmentation abnormalities in humans still require further validation through cellular and skin model studies, as well as clinical trials. This section specifically focuses on antioxidant‐mediated skin‐lightening agents that primarily target intracellular redox regulation within melanocytes.

*Salvia miltiorrhiza* volatile oil ameliorates melanin oxidative deposition through oxygen‐free radical scavenging [100], while Portulaca *oleracea* polysaccharide (VPOP3) exerts its effects via a dual mechanism involving both ROS inhibition and tyrosinase activity modulation [51]. Quantitative analyses demonstrated a significant correlation between the DPPH radical scavenging capacity (92.12%) and tyrosinase inhibition rate (70.38%) in selenium‐enriched Cardamine violifolia, confirming selenium's unique contribution to redox homeostasis [101]. Notably, the potent free radical scavenging capacity of flavonoids, attributed to their phenolic hydroxyl structures, was highlighted in comparative studies of five chrysanthemum varieties, where Bozhou chrysanthemum‐specific flavonoid metabolites (including apigenin and rutin) were speculated as key mediators of both intracellular radical elimination and tyrosinase inhibition [48], thereby establishing critical structure–activity relationships for bioactive compound screening. These results suggest that the phenolic hydroxyl groups in flavonoids may contribute to their antioxidant capacity. However, their specific effects on the skin require further validation through a combination of cellular experiments and skin models.

Aescin, a bioactive compound derived from Aesculus chinensis Bunge, can alleviate oxidative stress and autophagy by Keap1‐Nrf2 signaling, showing the potential as abundant sources of skin‐lightening agents [102].

### 3.7 Acceleration of Epidermal Turnover and Desquamation

Enhanced epidermal renewal has emerged as a novel therapeutic target for botanical skin‐lightening agents. *Solanum muricatum* (pepino) extract promotes melanin metabolism through improved cutaneous hydration [103]. *Portulaca oleracea* polysaccharide demonstrates cytoprotective effects against UVB‐induced keratinocyte apoptosis [51], findings that collectively suggest epidermal photodamage repair may emerge as a critical research frontier in dermal depigmentation strategies.

## 4 Discussion

Reviewing the development of skin‐lightening agents, it has gradually progressed from chemically synthesized skin‐lightening agents to plant‐based skin‐lightening agents. Although phenolic skin‐lightening agents, such as phenol, significantly reduce intracellular MITF in melanocytes and inhibit melanin‐related gene expression, they disrupt the skin's self‐protection against oxidative stress [104]. In contrast to synthetic counterparts, natural products exhibit distinct advantages characterized by multi‐component composition, polypharmacological targeting, and high chemical diversity. This synergistic potential is exemplified by the compound formulation Xinqibai extract (XQB)‐comprising seven botanicals (*Ampelopsis japonica*, *Paeonia lactiflora* , *Tribulus terrestris* , *Bletilla striata* , *Dictamnus dasycarpus*, *Poria cocos*, and *Atractylodes macrocephala*)‐which concurrently suppresses MITF (microphthalmia‐associated transcription factor) and TYR (tyrosinase) gene expression [92], demonstrating the therapeutic superiority of phytocomplex systems through coordinated multi‐target modulation. Moreover, the optimization of natural extract extraction methods leads to the enrichment of beneficial components and the removal of hazardous components. The aqueous extract of Angelica dahurica not only inhibits tyrosinase better than ethanol and ethyl acetate extracts but is also free of photosensitizing coumarins (Imperatorin and Isoimperatorin), a possible cause of allergic reactions [105]. The pharmacological effects of botanical extracts are critically influenced by extraction methodologies, as exemplified by *Salvia miltiorrhiza* derivatives. While both its volatile oil and ethanol extract demonstrate superior tyrosinase inhibition compared to arbutin, mechanistic analyses reveal distinct inhibitory patterns—the ethanol extract exhibits mixed‐type inhibition, whereas the volatile oil acts through competitive inhibition [100]. Notably, even within aqueous extracts of *S. miltiorrhiza* , significant functional divergence exists: total phenolic acids achieve 27% tyrosinase inhibition, in contrast to tanshinones, which show negligible activity [106], underscoring the critical role of phytochemical fractionation in bioactivity optimization. In summary, given the developmental complexity of natural skin‐lightening agents, adopting tailored development strategies for distinct plant types could facilitate their efficient utilization. Primarily, for plants demonstrating monocomponent bioactivity, development challenges predominantly focus on the extraction and purification of core constituents. Besides, when chemical synthesis or synthetic biological methodologies become applicable, particular attention must be paid to stereochemical integrity and bioactivity validation in vitro/in vivo. Ultimately, regarding plants requiring complex phytocompound matrices for efficacy, development priorities should emphasize the optimization of cultivation protocols for source plants and systematic monitoring of active compound profiles and secondary metabolite assemblies.

While current research predominantly focuses on terrestrial plants, marine organisms exhibit considerable potential as sources of skin‐lightening agents. Red algae polysaccharides and their degradation products have demonstrated tyrosinase inhibitory activity [107], with the Bangladeshi red seaweed *Hypnea musciformis* —particularly rich in phenolic compounds and flavonoids—showing notable antioxidant capacity. However, systematic investigations into marine‐derived natural skin‐lightening agents remain limited, primarily due to the structural complexity of their unique chemical constituents and technical challenges in sustainable procurement [108].

The development of botanical skin‐lightening agents has been revolutionized by the integration of multi‐disciplinary technological innovations, including structure–activity‐guided molecular modification, AI‐driven phytocompound screening platforms, and precision molecular docking systems for identifying novel tyrosinase inhibitory peptides—collectively enabling systematic exploration of plant‐derived skin‐lightening agents from virtual prediction to functional validation. Molecular docking has been applied to the development of tyrosinase inhibitors, while human tyrosinase is now commonly used to replace mushroom tyrosinase for molecular docking analysis [109]. Screening of activity components reveals 5‐hydroxyxanthotoxin and oxypeucedanin as the major components inhibiting tyrosine activity of Angelica dahurica extracts [105].

While developing novel plant‐derived skin‐lightening agents, safety profiling must be prioritized alongside efficacy evaluation. 4‐(4‐Hydroxyphenyl)‐2‐butanone (raspberry ketone, RK) has been implicated in chemical/occupational leukoderma pathogenesis, with mechanistic studies revealing RK's melanocytotoxicity stems from dual pathways: (1) covalent binding of RK‐derived quinones to thiol‐containing proteins and (2) pro‐oxidative activity of RK oligomers [110]. Notably, clinical cases of leukoderma have been reported following use of skin‐lightening creams containing diethylstilbestrol dipalmitate, *Glycyrrhiza* root extract, and *Mitracarpus scaber* extracts, highlighting potential risks of uncharacterized phytocomplexes [111]. Emerging solutions include a novel screening protocol differentiating phenolic tyrosinase inhibitors from leukoderma‐inducing catechols. This methodology rapidly assesses whether phenolic/catecholic compounds may trigger contact/occupational leukoderma through ortho‐quinone activation pathways, thereby enabling proactive safety evaluation during early‐stage development of botanical skin‐lightening agents [112].

## Author Contributions

Conceptualization, Lihao Gu; methodology, Siting Yang and Mingning Ding; software, Chenglong Ye and Jingning Shen; validation, Lihua Zhang and Anquan Yang; formal analysis, Siting Yang, Mingning Ding, and Chenglong Ye; investigation, Lihao Gu; resources, Lihao Gu; data curation, Jingning Shen; writing – original draft preparation, Mingning Ding, Chenglong Ye, and Jingning Shen; writing – review and editing, Siting Yang, Lihua Zhang, Anquan Yang and Lihao Gu; funding acquisition, Lihao Gu. All authors have read and agreed to the published version of the manuscript.

## Funding

This research was funded by the Zhejiang Provincial Leading Innovation and Entrepreneurship Team Project under the administration of the Science and Technology Department of Zhejiang Province (Grant No. 2024R01018). The article processing charge (APC) was funded by Osman Biological Co. Ltd.

## Ethics Statement

This study did not involve human participants or animals; therefore, ethical approval was not required.

## Conflicts of Interest

The authors declare no conflicts of interest.

## Data Availability

No new data were generated or analyzed in this study. All information discussed in this review is derived from previously published literature and is cited within the article.
